# HLA-DRA Gene Polymorphisms Are Associated with Graves' Disease as an Autoimmune Thyroid Disease

**DOI:** 10.1155/2022/6839634

**Published:** 2022-09-12

**Authors:** Peng Du, Jiaoli Zhu, Qiuming Yao, Tiantian Cai, Jianbin Xu, Yudie Fang, Yuqing Wu, Wen Zhang, Jin-an Zhang

**Affiliations:** ^1^Graduate School, Shanghai University of Traditional Chinese Medicine, Shanghai 201203, China; ^2^Department of Nephrology & Endocrinology, Suzhou Integrated Chinese and Western Medicine Hospital, Suzhou 215101, China; ^3^Department of Endocrinology & Rheumatology, Shanghai University of Medicine & Health Sciences Affiliated Zhoupu Hospital, Shanghai 201318, China; ^4^Department of Internal Medicine, Affiliated Maternity and Child Health Care Hospital of Nantong University, Nantong 226006, China; ^5^Shanghai University of Traditional Chinese Medicine, Shanghai 201203, China

## Abstract

**Background:**

Autoimmune thyroid disease (AITD), one of the most prevalent organ-specific autoimmune diseases, mainly includes Graves' disease (GD) and Hashimoto's thyroiditis (HT). This study was aimed at researching the association between AITD and single nucleotide polymorphisms (SNPs) of the HLA-DRA gene.

**Methods:**

Using Hi-SNP high-throughput sequencing technology, we detected the distribution of three SNPs (rs3177928, rs7197, and rs3129878) of HLA-DRA genotypes in 1033 AITD patients (634 GD and 399 HT ones) and 791 healthy volunteers in Chinese Han Population. Chi-square test, multivariate logistic regression, and haplotype analysis were performed by SPSS and Haploview software to analyze the relationship between HLA-DRA gene polymorphisms and AITD.

**Results:**

The results show that allele frequency and genotype distribution of rs3177928 and rs7197 were correlated with AITD and GD compared with the healthy control group, but not with HT. Rs3177928 and rs7197 were correlated with AITD and HT in the allele model, dominant model, and overdominant model before and after gender and age adjustment, but not with HT. In addition, we found that two loci (rs3177928 and rs7197) constituted a linkage disequilibrium (LD) region, and haplotype AA was associated with AITD and GD. However, we found no association between rs3129878 and AITD.

**Conclusion:**

Our study is the first to find that rs3177928 and rs7197 of HLA-DRA are significantly correlated with AITD and GD in the Chinese Han population. This will help further reveal the pathogenesis of AITD and provide new candidate genes for the prediction or treatment of AITD.

## 1. Introduction

Autoimmune thyroid disease (AITD) is one of the most prevalent organ-specific autoimmune diseases with a similar genetic and immunological basis in clinical practice. Clinically, Graves' disease (GD) and Hashimoto's thyroiditis (HT) are the most common, manifested as severe hyperthyroidism and hypothyroidism, both of which have lymphocyte infiltration and autoantibody production. The latest statistics show that the global prevalence of AITD is close to 5%, and the proportion of subclinical AITD with only positive antithyroid antibody but no symptoms will be higher [1, 2], especially in women aged 30 to 40. The prevalence ratio of AITD in women to men is between 5 : 1 and 10 : 1 [3].

The mechanism of inducing autoimmune attacks on the thyroid is still being explored, but a large number of studies have pointed out that the interaction between genetic susceptibility and environmental predisposing factors is the key cause of immune tolerance abnormalities and morbidity [4]. Human leukocyte class II antigens (HLA-II) can bind antigenic peptides and present them to T lymphocytes as cell surface receptors [5]. HLA-II gene polymorphisms are extremely important in regulating immune activity and not only determine the specificity of the antigen and the starting of the immune response but also affect the differentiation process of the T cell line in the thymus and control cytokine secretion, adjusting the strength of the immune response by cytokine gene on the haploid type. HLA susceptibility alleles can also contribute to GD/HT by the priority adjustment on the helper T cell (Th)2 (B lymphocyte stimulation) or Th1 (T lymphocyte-mediated injury) pathways, respectively. The main inducing factors of autoimmunity are genetic and environmental ones, and HLA susceptible alleles add up to the risk of infection with autoimmune diseases in a specific environment [6].

HLA-II genes are divided into subregions such as DR, DQ, DP, DO, DN, and DM. At present, the correlation between single nucleotide polymorphisms of HLA-DQ, HLA-DRB, and AITD has been confirmed [7–9]. In Japanese children, the frequencies of HLA-DRB1∗0405 and DQB1∗0401 were increased in the GD patients [10]. In the Greek population, the frequencies of DRB1∗0405, DQB1∗0201, DQB1∗0302, and DQA1∗0301 in HT patients were significantly higher than those in the normal population, and the frequency of HLA-DRB1∗07 was significantly lower [8]. A study in a Thai population found that the HLA-DRB1∗1602 allele and the closely linked haplotype DRB1∗1602-DQA1∗0102-DQB1∗0502 could serve as markers of genetic susceptibility to GD in Asian populations [7]. In addition, HLA-DRB3∗0101 was a susceptibility allele for HT, and DRB1∗0901, DRB1∗1001, and DRB4∗0101 were protective alleles for HT in the Jamaican population [9]. However, there are still few studies on the relationship between SNPs of HLA-DRA and AITD. This study was aimed at researching the relationship between HLA-DRA gene polymorphisms and AITD in the Chinese Han population. In addition, we analyzed the association between HLA-DRA genotype and clinical subtypes of AITD.

## 2. Methods

### 2.1. Subjects

A total of 1824 subjects were recruited, including 1033 AITD patients (243 males and 790 females, mean age of 41.71 ± 14.24) and 791 healthy controls (314 males and 477 females, mean age of 38.76 ± 10.53). AITD patients were divided into 634 GD (182 males and 452 females, mean age 41.09 ± 14.54 years) and 399 HT (61 males and 338 females, mean age 42.69 ± 13.70 years). All subjects were from Zhoupu Hospital Affiliated with Shanghai Health Medical College. Newly diagnosed GD patients included in the research fit the criteria for a GD diagnosis: characteristic clinical symptoms of hyperthyroidism, lab-confirmed hyperthyroidism, and a positive thyrotropin receptor antibody (TRAb) [11]. HT patients were identified on the basis of goiter and presence of thyroid peroxidase antibody (TPOAb) or thyroglobulin antibody (TgAb) with hypothyroidism or not. People with histories of other autoimmune and chronic disorders were eliminated from the control group. TPOAb, TgAb, TRAb, and other thyroid function indicators were determined by a highly sensitized and targeted immunochemiluminescence kit (Roche). All subjects were Han Chinese. Written informed consents were provided to all participants. The ethics committee of Shanghai Pudong New District Zhoupu Hospital approved this study.

### 2.2. Sample Collection and Genomic DNA Extraction

2 mL peripheral venous blood was taken from all subjects by venipuncture, and genomic DNA was extracted using the RelaxGene blood DNA system (Beijing Tiangen Biotechnology, China) according to the production guidelines. DNA concentration and purity of each sample were measured by a NanoDrop 2000 spectrophotometer (Thermo Science Company, Waltham, USA).

### 2.3. SNP Selection and Typing

We screened SNPs of the HLA-DRA gene from the HAPMAP-CHB database, which is strongly associated with other autoimmune diseases such as systemic lupus erythematosus and multiple sclerosis and finally screened three loci covering the entire region of the HLA-DRA gene, namely, rs3177928, rs7197, and rs3129878, to capture the most common variation of the gene. SNP screening was based on the three criteria as follows: (1) minor allele frequency (MAF) > 0.01, (2) Hardy-Weinberg equilibrium (HWE) > 0.05, and (3) logarithm of odds (LOD) > 3 : 0.

Multiplex PCR and high-throughput sequencing genotyping were used to detect the four SNPs. In short, primers were used to amplify DNA samples in a 10 *μ*L PCR reaction at 95°C for 15 min, followed by 5 cycles of 94°C for 30 s, 60°C for 4 min, 72°C for 30 s, 94°C for 30 s (10 cycles), 60°C for 1 min, and 72°C for 30 s. The following primer pairs were used: forward 5′-TGGTTGCTTATTATGTTCAGTTGG-3′ and reverse 5′-AAAGCAGAAGTTTCTTCAGTGATC-3′ for rs3177928, forward 5′-CTTCAACTCCTTGGTAACTATGTG-3′ and reverse 5′-TAGATGTTAGAGTACGGAGCAATC-3′ for rs7197, and Forward 5′-GTAGAAAAGTATTCTTCACCCAGC-3′ and reverse 5′-GCAAACACACACAAATATACTAGC-3′ for rs3129878. We repeated two negative reactions with water as the template for each PCR to ensure the quality of the genotyping process. The amplification products were purified, thinned, and randomly distributed. The genotyping was performed on the Illumina X-10 Platform (Illumina, USA) by using next-generation sequencing technology. Then, we analyzed samples with inconsistent genotyping results by Sanger sequencing and only further analyzed samples exceeding the 95% quality control level.

### 2.4. Data Analysis

SPSS software (Version 23.0, IBM, Chicago, USA) was used for all statistical analyses. The classification data were presented as frequency or percentage, and the quantitative data were presented as mean ± standard deviation (mean ± SD). The allele/genotype frequency of SNPs was analyzed by the Pearson chi-square test. Multivariate logistic regression analysis was performed on confounding factors such as gender and age, and *P* values and OR values were calculated before and after adjustment. Allele, dominant, overdominant, recessive, and additive models were used to analyze the relationship between HLA-DRA gene polymorphisms and AITD. Haploview 4.2 software (Broad Institute, Cambridge, MA, USA) was used to assess HWE and haplotype. *P* values less than 0.05 were deemed to be statistically significant.

## 3. Results

### 3.1. Clinical Data Analysis

Clinical data of all subjects are shown in Table 1. A total of 1033 AITD patients investigated in this study consisted of 634 patients with GD (28.7% males and 71.3% females, average age of 41.09 ± 14.54 years) and 399 patients with HT (15.3% males and 84.7% females, average age of 42.69 ± 13.70 years). Among the patients, there were 196 cases (19.0%) with family history, 101 cases (9.8%) with ophthalmopathy, and 63 cases (6.1%) with the age of onset ≤ 18 years. Among the GD patients, there were 133 cases of family history, 98 cases of eye disease, and ≤52 cases with the age of onset of 18 years (21.0%, 15.5%, and 8.2%, respectively). The HT group consisted of 63 cases of family history, 3 cases of eye disease, and 11 cases of onset age ≤ 18 years (15.8%, 0.8%, and 2.8%, respectively).

### 3.2. Allele and Genotype Analysis

The allele frequency and genotype distribution of *HLA-DRA* gene polymorphisms are shown in Table 2. For rs3177928 and rs7197, the frequency of allele A in the AITD and GD groups was lower than that in the control group (1.4%/1.1% vs. 3.0%, 1.4%/1.0% vs. 2.6%, respectively; *P* < 0.01). The rs3177928 and rs7197 genotype distributions of the healthy control group were different from those of AITD cases (with *P* values of 0.002 and 0.012, respectively) and the GD group (with *P* values of 0.001 and 0.004, respectively). Compared with the healthy control group, there was no significant difference in allele frequency and genotype distribution of rs3129878 locus of HLA-DRA gene in AITD, GD, and HT groups, with *P* values greater than 0.05. In Table 3, before adjusting for confounding factors (age and gender), there were significant differences in the allele model, dominant model, and overdominant model of rs3177928 in patients with AITD compared with healthy controls. OR values were 0.47 (95% CI 0.29-0.74, *P* = 0.001), 0.47 (95% CI 0.29-0.75, *P* = 0.001), and 0.48 (95% CI 0.30-0.77, *P* = 0.002), respectively. In addition, compared with healthy controls, OR values of the rs7197 allele model, dominant model, and overdominant model in AITD patients were 0.52 (95% CI 0.32-0.84, *P* = 0.007), 0.52 (95% CI 0.32-0.86, *P* = 0.009), and 0.54 (95% CI 0.33-0.88, *P* = 0.013), respectively; the differences were statistically significant. Multivariate logistic regression analysis after age and gender adjustment showed that rs3177928 and rs7197 were still significantly different (*P* < 0.05). In all gene association analysis models, there was no significant difference between AITD patients and healthy controls in rs3129878 of HLA-DRA before and after adjusting for age and gender (*P* > 0.05) (Table 3).

As shown in Table 4, rs3129878 of HLA-DRA in the GD group and control group showed no significant differences in allele model, dominant model, and overdominant model before and after adjusting for gender and age (*P* > 0.05). However, rs3177928 and rs7197 were significantly correlated with GD in the three models before and after adjusting for confounding factors (age and gender) (*P* < 0.01).  Table 5 shows that rs3129878 had no significant correlation with HT in the three models before and after adjusting for confounding factors (age and gender) (*P* > 0.05).

In addition, rs3177928, rs7197, and rs3129878 of HLA-DRA in the AITD/GD/HT group and control group showed no significant differences in the recessive model and additive model before and after adjusting for gender and age (data shown in Table 7/8/9 in the supplementary material (available here)).

### 3.3. Correlation between Genotype and Clinical Subtype

We also analyzed the relationship between genotype and clinical phenotype, regarding (1) age of onset in patients with AITD (≤18 years or ≥19 years); (2) ophthalmopathy in patients with GD (this was defined in terms of the clinical manifestations of proptosis, photophobia, excessive tearing, spontaneous post bulbar pain, eye movement pain, and diplopia [12]); and (3) hypothyroidism or nonhypothyroidism in patients with HT. The distribution of alleles or genotypes of the three SNPs of the HLA-DRA gene was not associated with the three clinical phenotypes (data not shown).

### 3.4. Haplotype Analysis

**Figure 1:**
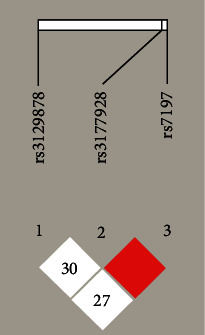
Linkage disequilibrium (LD) block in HLA-DRA gene shown by Haploview software.

Results using the Haploview software showed that there was an LD region consisting of two loci (rs3177928 and rs7197) (Figure 1) and three main haplotypes (GG, AA, and AG). As shown in Table 6, the AA haplotype was associated with AITD and GD in our population (*P* < 0.05).

## 4. Discussion

In this research, we revealed that the allele frequency and genotype distribution of rs3177928 and rs7197 of the HLA-DRA gene were correlated with AITD and GD, and the allele model, dominant model, and overdominant model of rs3177928 and rs7197 in the AITD and GD groups differed significantly from those in the control group. The association of a gene with a complex disease can be more fully demonstrated by haplotype analysis. We found strong LD in two loci (rs3177928 and rs7197) between patients and controls, and further analysis showed that haplotype AA was correlated with AITD and GD. HLA-DRA gene polymorphisms were significantly correlated with AITD and GD; however, we did not find a correlation between HLA-DRA gene polymorphisms and HT. Although HT and GD both belong to AITD and have a similar genetic and immunological basis, their etiology and pathogenesis are not completely the same [13]. The different associations of HLA-DRA gene polymorphisms with GD and HT are theoretically possible.

HLA-DR belongs to the major histocompatibility complex II receptor, which is a transmembrane heterodimer composed of *α* and *β* glycoprotein chains. The *α* and *β* chains are encoded by different genes whose transcriptional level expression is precisely controlled [14]. HLA-DR is expressed by antigen-presenting cells such as monocytes, B cells, and dendritic cells but can also be generated in nonspecialized APCs such as desmocytes, endothelial cells, and activated T lymphocytes under an inflammatory state [15]. HLA-DRA gene is located on human autosomal 6P21.32 and contains five exons, encoding the *α* chain of the HLA-DR antigen molecule and forming a heterodimer with the *β* chain encoded by the HLA-DRB gene, which plays an important role in CD4+ T cell-mediated immune response [16]. CD4+ T cells are the main effector cells of the adaptive immune response. CD4+ T lymphocytes can be divided into different subsets, namely, Th1, Th2, Th17, and regulatory T cells (Treg), according to differences in cell differentiation and phenotype [17]. The skew ratio of Th1/Th2 cells, overactivated Th17 cells, and the abnormal inhibitory effect of Treg cells can help clarify the pathogenesis of AITD [18–21].

Current studies have shown that HLA class II gene polymorphisms are correlated with AITD [22–24]. As for the genetic susceptibility mechanisms of HLA and AITD, one explanation is that HLA's own structure and function are related to the occurrence of disease. Another explanation is that additional HLA-associated genes are involved in AITD. One theory is that individuals with specific genetic qualities would be affected by environmental factors (such as bacteria, viruses, and iodine) and stress state (such as trauma); then, the HLA antigen combined with the specific antigen peptide incorrectly presents the thyroid tissue antigen to T lymphocytes, and combines with CD4+/CD8+ T lymphocyte receptors, activates T lymphocytes, produces cytokines to regulate immune response, and causes the occurrence of AITD through activation of B lymphocytes to produce antibodies [25]. In addition, the distal functional domain of the extracellular region of HLA antigens can bind to antigens and contain disease-related key amino acid residues, whose changes can affect the characteristics of the interaction between HLA antigens and T cell receptors, and therefore alter the immune response in the presence of foreign/autoantigens [26]. It is widely believed that the occurrence of AITD is in line with the multifactorial genetic model. When environmental factors and genetic factors interact with each other and the risk exceeds the theoretical threshold, it presents as a disease state clinically. Some genetic factors affect the expression of HLA genes encoding immune response proteins [27].

HLA class II genes are highly polymorphic, but HLA-DRA gene polymorphisms are highly conserved and are more likely to cause functional changes than other MHC class II genes [28] and have been considered a target of immune-mediated diseases [29]. The relationship between HLA-DRA gene polymorphisms and autoimmune diseases has been studied [30]. HLA-DRA is shown to be a susceptibility gene for multiple sclerosis in the Australian population. Polymorphisms of rs4935356, rs3177928, and rs7197 of the HLA-DRA gene have been associated with multiple sclerosis in the Iranian population [31]. In addition, HLA-DRA gene polymorphisms are associated with the clinical course and long-term prognosis of ulcerative colitis [32].

Recent studies have shown that SNP at rs3177928 is associated with sarcoidosis [33], as well as lipid metabolism and inflammatory mechanisms [34–37]. More recently, rs7197 SNP has been found to be closely related to the antibody response to viral elements such as capsid antigen of Epstein-Barr virus, and it is speculated that rs7197 genetic variation may play a role in disease susceptibility through immune-mediated specific microbial genes [31]. Combining our study with previous in vitro and in vivo evidence that Epstein-Barr virus infection is associated with TRAb production and GD occurrence [38, 39], it is reasonable to infer that there is a natural association between rs3177928, rs7197 polymorphisms, and AITD development.

To our knowledge, this is the first study to investigate the association of HLA-DRA SNPs with AITD, thus providing new insights into the role of HLA-DRA in AITD. Still, this study has some limitations. First of all, our study was limited to the Chinese Han population and may have a racial bias. Secondly, this study only evaluated the relationship between HLA-DRA gene polymorphisms and AITD susceptibility, and its molecular mechanism needs to be further clarified. Third, although the differences were statistically significant, there would be some limitations in genotyping data due to the very small frequency of minor alleles.

In summary, we found for the first time that two SNPs of HLA-DRA were correlated with GD, but not with HT in the Chinese Han population. Although both GD and HT were involved in the infiltration of thyroid lymphocytes and the production of autoantibodies, there were some differences in their pathogenesis. Larger sample size is needed to further confirm the study's conclusions.

## Figures and Tables

**Table 1:** Clinical data of all subjects.

*N*	AITD	GD	HT	Controls
	1033	634	399	791
Gender				
Male	243 (23.5)	182 (28.7)	61 (15.3)	314 (39.7)
Female	790 (76.5)	452 (71.3)	338 (84.7)	477 (60.3)
Age (mean ± SD)	41.71 ± 14.24	41.09 ± 14.54	42.69 ± 13.70	38.76 ± 10.53
Family history				
(+)	196 (19.0)	133 (21.0)	63 (15.8)	—
(-)	837 (81.0)	501 (79.0)	336 (84.2)	—
Ophthalmopathy				
(+)	101 (9.8)	98 (15.5)	3 (0.8)	—
(-)	932 (90.2)	536 (84.5)	396 (99.2)	—
Onset of age				
≤18 years	63 (6.1)	52 (8.2)	11 (2.8)	—
≥19 years	970 (93.9)	582 (91.8)	388 (77.2)	—

AITD: autoimmune thyroid disease; GD: Graves' disease; HT: Hashimoto's thyroiditis.

**Table 2:** Allele frequencies and genotype distribution of HLA-DRA polymorphisms in AITD patients and controls.

Gene/SNP	Controls	AITD	*P* value (OR 95% CI)	GD	*P* value (OR 95% CI)	HT	*P* value (OR 95% CI)
*HLA-DRA*	*N* (%)	*N* (%)	AITD vs. control	*N* (%)	GD vs. control	*N* (%)	HT vs. control
rs3177928							
A	47 (3.0)	29 (1.4)	0.001	14 (1.1)	<0.001	15 (1.9)	0.134
G	1535 (97.0)	2037 (98.6)	0.465 (0.291-0.742)	1254 (98.9)	0.365 (0.200-0.665)	783 (98.1)	0.626 (0.348-1.126)
AA	1 (0.1)	0 (0)		0 (0)		0 (0)	
AG	45 (5.7)	29 (2.8)	0.002	14 (2.2)	0.001	15 (3.8)	0.270
GG	745 (94.2)	1004 (97.2)		620 (97.8)		384 (96.2)	
rs7197							
A	41 (2.6)	28 (1.4)	0.007	13 (1.0)	0.002	15 (1.9)	0.279
G	1541 (97.4)	2038 (98.6)	0.516(0.318-0.839)	1255 (99.0)	0.389 (0.208-0.730)	783 (98.1)	0.720 (0.396-1.309)
AA	1 (0.1)	0 (0)		0 (0)		0 (0)	
AG	39 (4.9)	28 (2.7)	0.012	13 (2.1)	0.004	15 (3.8)	0.589
GG	751 (95.0)	1005 (97.3)		621 (97.9)		384 (96.2)	
rs3129878							
A	1132 (71.6)	1508 (73.0)	0.336	929 (73.3)	0.311	579 (72.6)	0.608
C	450 (28.4)	558 (27.0)	0.931 (0.804-1.077)	339 (26.7)	0.918 (0.778-1.803)	219 (27.4)	0.951 (0.787-1.151)
AA	398 (50.3)	532 (51.5)		330 (52.1)		202 (50.6)	
AC	336 (42.5)	444 (43.0)	0.334	269 (42.4)	0.416	175 (43.9)	0.531
CC	57 (7.2)	57 (5.5)		35 (5.5)		22 (5.5)	

AITD: autoimmune thyroid disease; GD: Graves' disease; HT: Hashimoto's thyroiditis.

**Table 3:** Odds ratios (ORs) of the associations of three polymorphisms in the HLA-DRA gene with AITD before and after adjusting for confounders (age and gender).

Comparison models	Unadjusted estimates		Adjusted estimates^∗^	
	OR (95% CI)	*P* values	OR (95% CI)	*P* values
rs3177928				
Allele model	0.47 (0.29-0.74)	0.001	0.44 (0.27-0.70)	<0.001
Dominant model	0.47 (0.29-0.75)	0.001	0.44 (0.27-0.71)	<0.001
Overdominant model	0.48 (0.30-0.77)	0.002	0.45 (0.28-0.74)	0.0012
rs7197				
Allele model	0.52 (0.32-0.84)	0.007	0.49 (0.30-0.79)	0.0036
Dominant model	0.52 (0.32-0.86)	0.009	0.49 (0.30-0.81)	0.005
Overdominant model	0.54 (0.33-0.88)	0.013	0.51 (0.31-0.84)	0.0079
rs3129878				
Allele model	0.93 (0.80-1.08)	0.336	0.96 (0.82-1.12)	0.63
Dominant model	0.95 (0.79-1.15)	0.616	1.00 (0.82-1.21)	0.98
Overdominant model	1.02 (0.85-1.23)	0.83	1.05 (0.87-1.28)	0.59

AITD: autoimmune thyroid disease; OR: odds ratio; 95% CI: 95% confidence interval. ^∗^Age and gender were adjusted in the multivariate logistic regression analyses.

**Table 4:** Odds ratios (ORs) of the associations of three polymorphisms in the HLA-DRA gene with GD before and after adjusting for confounders (age and gender).

Comparison models	Unadjusted estimates		Adjusted estimates^∗^	
	OR (95% CI)	*P* values	OR (95% CI)	*P* values
rs3177928				
Allele model	0.37 (0.20-0.67)	<0.001	0.35 (0.19-0.65)	<0.001
Dominant model	0.37 (0.20-0.67)	<0.001	0.35 (0.19-0.65)	<0.001
Overdominant model	0.37 (0.20-0.69)	<0.001	0.36 (0.20-0.67)	<0.001
rs7197				
Allele model	0.39 (0.21-0.74)	0.0018	0.38 (0.20-0.71)	0.0012
Dominant model	0.39 (0.21-0.74)	0.0021	0.38 (0.20-0.72)	0.0015
Overdominant model	0.40 (0.21-0.76)	0.003	0.39 (0.21-0.74)	0.0023
rs3129878				
Allele model	0.91 (0.77-1.08)	0.30	0.93 (0.78-1.11)	0.41
Dominant model	0.93 (0.76-1.15)	0.52	0.96 (0.77-1.18)	0.67
Overdominant model	1.00 (0.81-1.23)	0.99	1.02 (0.82-1.26)	0.88

GD: Graves' disease; OR: odds ratio; 95% CI: 95% confidence interval. ^∗^Age and gender were adjusted in the multivariate logistic regression analyses.

**Table 5:** Odds ratios (ORs) of the associations of three polymorphisms in the HLA-DRA gene with HT before and after adjusting for confounders (age and gender).

Comparison models	Unadjusted estimates		Adjusted estimates^∗^	
	OR (95% CI)	*P* values	OR (95% CI)	*P* values
rs3177928				
Allele model	0.63 (0.35-1.13)	0.11	0.60 (0.33-1.09)	0.084
Dominant model	0.63 (0.35-1.15)	0.12	0.61 (0.33-1.12)	0.10
Overdominant model	0.65 (0.36-1.18)	0.14	0.63 (0.34-1.17)	0.13
rs7197				
Allele model	0.72 (0.40-1.31)	0.27	0.67 (0.36-1.24)	0.19
Dominant model	0.73 (0.40-1.34)	0.31	0.69 (0.37-1.29)	0.23
Overdominant model	0.75 (0.41-1.38)	0.35	0.72 (0.38-1.35)	0.29
rs3129878				
Allele model	0.95 (0.78-1.15)	0.60	1.04 (0.85-1.28)	0.71
Dominant model	0.99 (0.78-1.26)	0.92	1.08 (0.84-1.40)	0.54
Overdominant model	1.06 (0.83-1.35)	0.65	1.11 (0.86-1.43)	0.43

HT: Hashimoto's thyroiditis; OR: odds ratio; 95% CI: 95% confidence interval. ^∗^Age and gender were adjusted in the multivariate logistic regression analyses.

**Table 6:** Distribution of haplotypes for HLA-DRA region polymorphisms in AITD, GD, HT patients, and control groups.

Haplotypes	Frequencies of haplotypes				*P* value; OR (95% CI)		
	NC	AITD	GD	HT	AITD vs. NC	GD vs. NC	HT vs. NC
GG	0.9703	0.986	0.989	0.9812	—	—	—
AA	0.0259	0.0136	0.0103	0.0188	0.0074; 0.52 (0.32-0.84)	0.0034; 0.39 (0.21-0.73)	0.27; 0.72 (0.40-1.30)
AG	0.0038	<0.001	<0.001	0	0.053; 0.20 (0.02-1.67)	0.14; 0.20 (0.02-1.67)	—

AITD: autoimmune thyroid disease; GD: Graves' disease; HT: Hashimoto's thyroiditis.

## Data Availability

The authors confirm that the data supporting the findings of this study are available within the article.

## Supplementary Materials

Supplementary Materials The differences between AITD/GD/HT group and the control group in rs3177928, rs7197, and rs3129878 before and after adjusting for confounding factors (age and gender) were analyzed by recessive model and additive model. These analyses are shown in Table 7/8/9.
