# Supplementary material for: HLA-DRA Gene Polymorphisms Are Associated with Graves' Disease as an Autoimmune Thyroid Disease
Source: Biomed Res Int. 2022 Sep 12;2022:6839634. doi: 10.1155/2022/6839634 (PMC9484895; doi:10.1155/2022/6839634)
Supplement: Supplementary Materials — The differences between AITD/GD/HT group and the control group in rs3177928, rs7197, and rs3129878 before and after adjusting for confounding factors (age and gender) were analyzed by recessive model and additive model. These analyses are shown in Table 7/8/9. [file 6839634.f1.doc]

**TABLE 7** Odds ratios (*OR*s) of the associations of three polymorphisms in the HLA-DRA gene with AITD before and after adjusting for confounders (age and gender).

| Comparison models | Unadjusted estimates | | Adjusted estimates* | |
| --- | --- | --- | --- | --- |
|  | *OR* (95% *CI*) | *P* values | *OR* (95% *CI*) | *P* values |
| rs3177928 |  |  |  |  |
| recessive model | — | 0.20 | — | 0.30 |
| additive model | — | 0.43 | — | 1.00 |
| rs7197 |  |  |  |  |
| recessive model | — | 0.20 | — | 0.30 |
| additive model | — | 0.43 | — | 1.00 |
| rs3129878 |  |  |  |  |
| recessive model | 0.75 (0.51-1.10) | 0.14 | 0.72 (0.47-1.11) | 0.14 |
| additive model | 0.75 (0.51-1.10) | 0.14 | 0.80 (0.54-1.20) | 0.28 |

AITD: autoimmune thyroid disease; OR: odds ratio; 95% CI: 95% confidence interval; *age and gender were adjusted in the multivariate logistic regression analyses.

**TABLE 8** Odds ratios (*OR*s) of the associations of three polymorphisms in the HLA-DRA gene with GD before and after adjusting for confounders (age and gender).

| Comparison models | Unadjusted estimates | | Adjusted estimates* | |
| --- | --- | --- | --- | --- |
|  | *OR* (95% *CI*) | *P* values | *OR* (95% *CI*) | *P* values |
| rs3177928 |  |  |  |  |
| recessive model | — | 0.28 | — | 0.41 |
| additive model | — | 0.36 | — | 1.00 |
| rs7197 |  |  |  |  |
| recessive model | — | 0.28 | — | 0.41 |
| additive model | — | 0.36 | — | 1.00 |
| rs3129878 |  |  |  |  |
| recessive model | 0.75 (0.49-1.16) | 0.20 | 0.68 (0.42-1.12) | 0.12 |
| additive model | 0.74 (0.47-1.16) | 0.22 | 0.76 (0.48-1.20) | 0.23 |

GD: Graves’ disease; OR: odds ratio; 95% CI: 95% confidence interval; *age and gender were adjusted in the multivariate logistic regression analyses.

**TABLE 9** Odds ratios (*OR*s) of the associations of three polymorphisms in the HLA-DRA gene with HT before and after adjusting for confounders (age and gender).

| Comparison models | Unadjusted estimates | | Adjusted estimates* | |
| --- | --- | --- | --- | --- |
|  | *OR* (95% *CI*) | *P* values | *OR* (95% *CI*) | *P* values |
| rs3177928 |  |  |  |  |
| recessive model | — | 0.37 | — | 0.30 |
| additive model | — | 0.47 | — | 1.00 |
| rs7197 |  |  |  |  |
| recessive model | — | 0.37 | — | 0.30 |
| additive model | — | 0.48 | — | 1.00 |
| rs3129878 |  |  |  |  |
| recessive model | 0.75 (0.45-1.25) | 0.26 | 0.91 (0.54-1.55) | 0.73 |
| additive model | 0.76 (0.45-1.28) | 0.30 | 0.99 (0.57-1.72) | 0.97 |

HT: Hashimoto’s thyroiditis; OR: odds ratio; 95% CI: 95% confidence interval; *age and gender were adjusted in the multivariate logistic regression analyses.
